# Two Cases of Periprosthetic Atypical Femoral Fractures in Patients on Long-Term Bisphosphonate Treatment

**DOI:** 10.1155/2019/9845320

**Published:** 2019-03-03

**Authors:** Takanori Miura, Hiroaki Kijima, Takayuki Tani, Toshihito Ebina, Naohisa Miyakoshi, Yoichi Shimada

**Affiliations:** ^1^Department of Orthopedic Surgery, Kakunodate General Hospital, 3-Iwase Kakunodatemachi, Senboku, Akita 014-0394, Japan; ^2^Department of Orthopedic Surgery, Akita University Graduate School of Medicine, 1-1-1 Hondo, Akita 010-8543, Japan; ^3^Akita Hip Research Group (AHRG), 1-1-1 Hondo, Akita 010-8543, Japan

## Abstract

The current definition of atypical femoral fractures (AFFs) excludes periprosthetic fractures. However, a few cases of bisphosphonates (BPs) -associated periprosthetic atypical femoral fractures (PAFFs) have been reported in the literature. Here, we report two rare cases of PAFFs that fulfilled the major criteria for AFFs in patients with prolonged use of BPs. Both cases progressed to a complete fracture with minor trauma from an incomplete fracture at the distal tip of the well-fixed femoral stem. The femoral stem effect on lateral femoral cortical bone, together with the decreased bone elastic resistance induced by BPs, was considered the cause of onset. In each case, we performed open reduction and internal fixation using a locking plate with cable grip and postoperatively prescribed teriparatide and low-intensity pulsed ultrasound (LIPUS). Both cases had a good clinical course. However, as conservative treatment was not effective in these cases, treatment such as non-weight-bearing exercises during hospitalization or prophylactic surgery may be necessary. Further studies are needed to determine the optimal treatment strategy.

## 1. Introduction

Atypical femoral fractures (AFFs) are stress or insufficiency fractures induced by low-energy trauma or no trauma and present specific X-ray findings. Interestingly, AFFs have emerged as potential complications of bisphosphonates (BPs) use over the past decade. The American Society for Bone and Mineral Research (ASBMR) published a Task Force report on AFFs in 2014 [1]. Although the definition of AFFs in the report excluded periprosthetic fractures [1], several case reports have described bisphosphonate-associated AFFs occurring around the stem of the femur [2–14]. Despite awareness of periprosthetic AFFs (PAFFs), their characteristics, diagnostic criteria, and treatment have not been determined [13]. Here, we report two rare cases of PAFFs associated with prolonged use of BPs that fulfill the ASBMR major criteria for AFFs.

## 2. Case Presentation

### 2.1. Case 1

**Figure 1:**
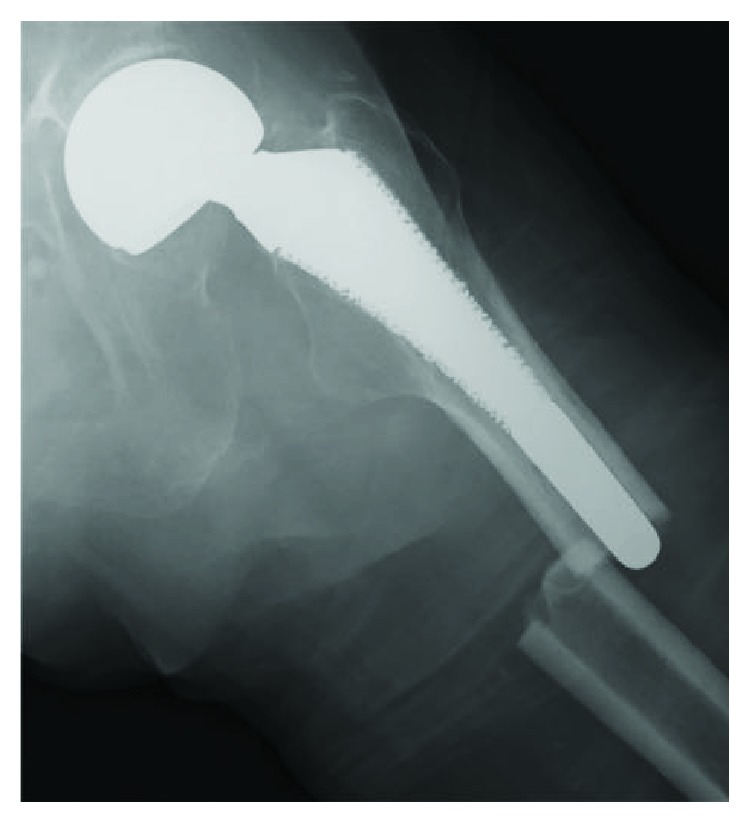
Anteroposterior pelvis radiograph showing a noncomminuted transverse fracture located at the tip of the stem with localized periosteal thickening of the lateral cortex, or a “beak sign,” of the left femur.

**Figure 2:**
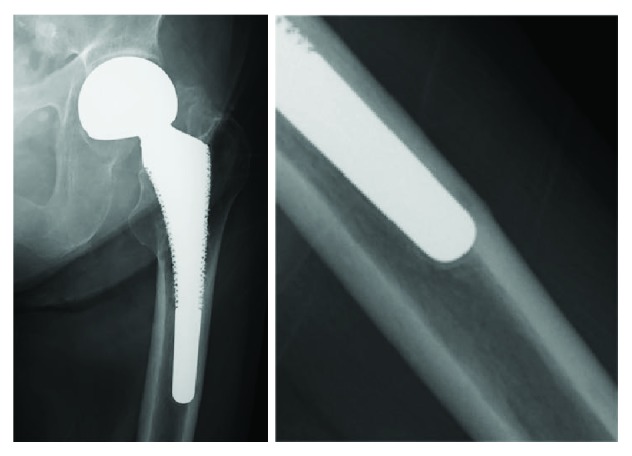
Anteroposterior pelvis radiograph taken two years prior showed that the femoral stem was in contact with the lateral side of the femur; there was no evidence of fractures and localized periosteal thickening of the lateral cortex slightly emerging in the same area.

**Figure 3:**
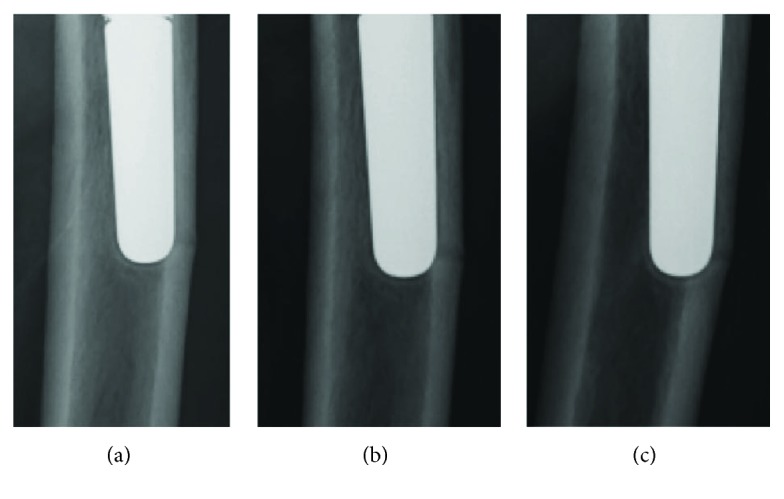
Magnified anteroposterior radiographs of the fracture line, showing gradual clarification of the fracture at six-month follow-up (a), one-year follow-up (b), and two-year follow-up (c).

**Figure 4:**
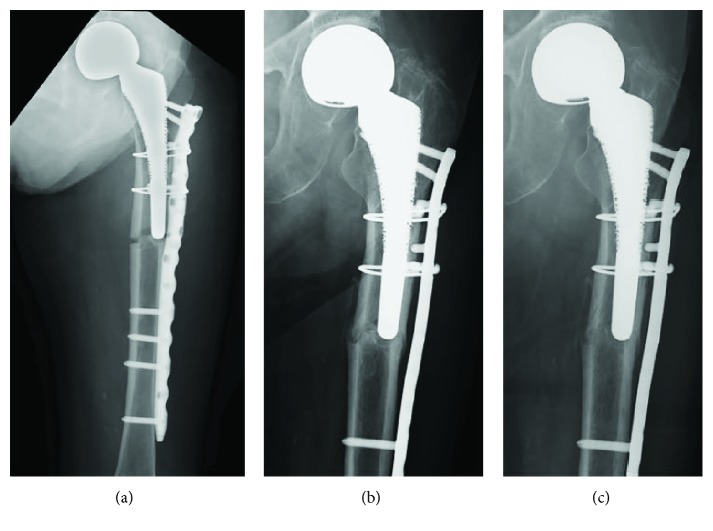
Anteroposterior radiographs showing fixation of the periprosthetic fracture using a cable-plate device (a) after the operation, (b) at one-year follow-up, and (c) at two-year follow-up.

An 81-year-old woman with a left bipolar hemiarthroplasty performed 10 years previously presented with a left femoral shaft fracture that occurred without trauma (Figure 1). Her femur broke while she was standing, and then, she fell down. She had visited our hospital with new-onset left thigh pain two years prior to this episode. Radiographs showed no evidence of a fracture, but slight localized periosteal thickening of the lateral cortex was observed (Figure 2). In addition, she had undergone osteoporosis treatment and had been taking alendronate for more than five years. She was instructed to discontinue alendronate, prescribed vitamin D, and continue with limited weight bearing with cane. After 6 months (1.5 years before the injury), the fracture line became clearer; however, the pain had disappeared. Thus, the conservative treatment was continued. At 12 months (1 year before the injury), a fracture line was visible; however, there was no complaint of pain. However, at 24 months (10 days before the injury), the patient reported pain (Figure 3). After the injury, radiographs showed a noncomminuted transverse fracture located at the tip of the stem with localized periosteal thickening of the lateral cortex or a “beak sign”; we judged Vancouver type B1 periprosthetic fracture (Figure 1). The fracture was complete, extending through both cortices. We used a locking plate with cable grip to perform open reduction and internal fixation (Figure 4(a)). Postoperatively, the patient was allowed non-weight-bearing exercise. She was prescribed weekly subcutaneous injection of 56.5 *μ*g teriparatide and low-intensity pulsed ultrasound (LIPUS). One year later, radiographs revealed nonunion (Figure 4(b)). At the two-year follow-up, complete bone union was achieved (Figure 4(c)). At the most recent follow-up (three years), there was no tenderness over the fracture site and radiographs revealed no displacement or loosening of the implants.

### 2.2. Case 2

**Figure 5:**
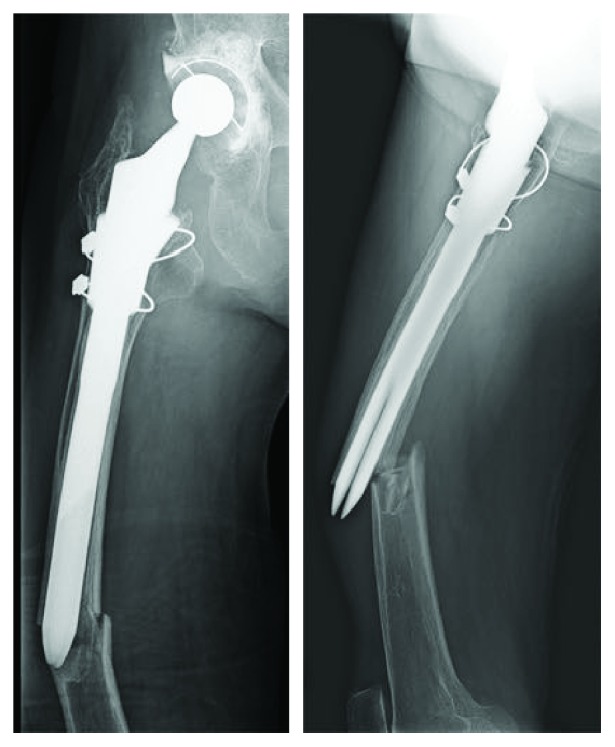
Radiograph showing a noncomminuted transverse fracture located at the tip of the well-fixed femoral stem with a medial spike, third bone fragment.

**Figure 6:**
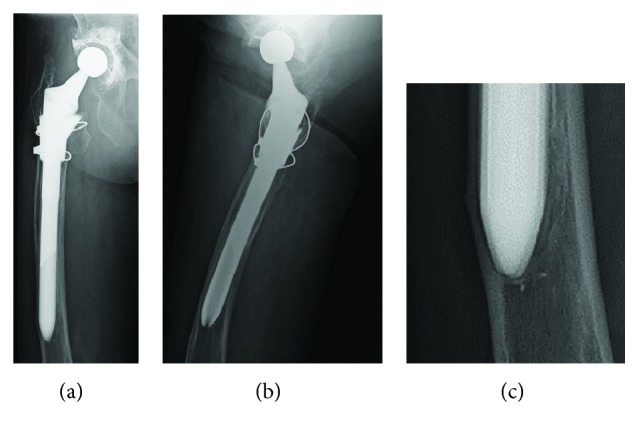
(a) Anteroposterior radiograph, (b) mediolateral radiograph, and (c) magnified anteroposterior radiographs of the fracture line. Five months before hospitalization, the cortical bone was thinning slightly in proximity to the tip of the stable femoral stem and the transverse lucency was admitted on the outside.

**Figure 7:**
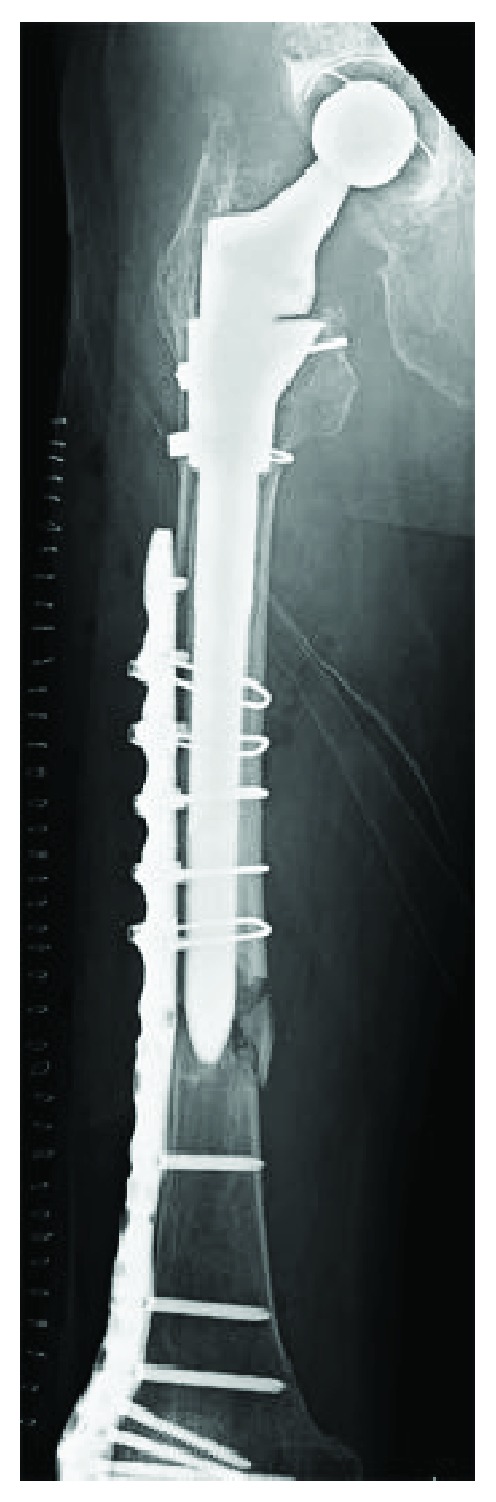
Anteroposterior radiograph showed fixation of the periprosthetic fracture using a cable-plate device following the operation.

An 85-year-old woman visited our hospital due to a right femur fracture that occurred after minor trauma as a result of having fallen down while walking. She had received a right total hip arthroplasty 18 years earlier due to rapidly destructive coxopathy and a revision arthroplasty 9 years earlier because of the loosening of the femoral stem. In addition, the patient had been taking alendronate for more than five years. Radiographs showed localized thickening of the lateral femoral cortical bone and complete transverse fracture with internal spikes. In addition, a third bone fragment was seen, showing the same findings as the atypical femoral fracture. We judged Vancouver type B1 periprosthetic fracture (Figure 5). In the radiographs taken five months before hospitalization, the cortical bone appeared to be thinning slightly in proximity to the tip of the stable femoral stem and the transverse lucency was emitted on the outside (Figure 6), which resulted in a complete fracture from the incomplete fracture. We used a locking plate with cable grip to perform open reduction and internal fixation (Figure 7). Bisphosphonate use was discontinued and prescribed weekly subcutaneous injections of 56.5 *μ*g teriparatide and LIPUS. The patient was allowed non-weight-bearing exercise. At the follow-up three months after surgery, the patient was using a wheelchair without pain and a radiograph revealed no displacement or loosening of the implants.

## 3. Discussion

We reported two rare cases of PAFFs that fulfill the major criteria for AFFs and that involve prolonged use of BPs. Both cases progressed to a complete fracture with minor trauma from an incomplete fracture at the stem tip of the well-fixed femoral stem.

The 2013 ASBMR diagnostic criteria for AFFs require the presence of at least 4 of the 5 major criteria. The minor features include a generalized increase in cortical thickness of the femoral diaphysis, prodromal symptoms such as thigh pain and bilateral incomplete or complete femoral diaphysis fracture, and delayed fracture healing. The AFF definition excludes periprosthetic and pathologic fractures [1]. However, the characteristic features of atypical fractures have been recently reported in the so-called PAFFs [2–14]. A review of the literature identified 13 articles reporting 26 cases of PAFFs that were not randomized controlled trials or studies comparing nonoperative versus operative treatment. These studies are summarized in Table 1.

Associations have been reported between AFFs and BPs, steroids, rheumatoid arthritis, femoral lateral bowing, and diabetes mellitus [1, 15]. There is an especially strong association between the long-term use of antiresorptive agents (such as BPs) and severe suppression of bone turnover (SSBT) [16]. Such agents result in decreased bone remodeling and low turnover, thereby increasing bone micro damage accumulation and decreasing bone healing capacity, resulting in the deterioration of bone quality. A multifactorial etiology, including poor bone quality due to mutual interactions and mechanical stress, appears to be responsible for the occurrence of AFFs [17]. By contrast, the factors of typical periprosthetic fractures are said to be patient- and implant-related, such as femoral stem loosening, malalignment, osteolysis, cortical defects, incomplete cement mantle, and stem tip impingement [18]. The two cases reported here fulfill the major criteria for AFFs, involve prolonged use of BPs, and have a history of incomplete femoral fracture. Based on these factors, we diagnosed PAFFs. Uncemented femoral stems inserted in varus alignment are known to have a significant negative influence on the survival of the femoral stem [19]. However, varus alignment does not affect osteolysis and radiolucent lines [19] or cortical atrophy [20]. Since femoral stems affect the lateral femoral cortical bone, together with the decrease in bone elastic resistance induced by BPs, they can produce periprosthetic femoral fractures even in well-positioned stem [14]. In our cases, it was thought that PAFFs developed due to mechanical stress, such as femoral stem contacting the lateral cortical bone, and SSBT caused by long-term oral administration of BPs.

Although surgical treatment is generally performed for complete PAFFs, the best method of treatment for incomplete PAFFs is controversial. Curtin et al. reported three cases of incomplete PAFFs treated by discontinuing BPs and limiting weight bearing; later, teriparatide was added to two cases [3]. Cross et al. reported incomplete PAFFs treated by discontinuing BPs, starting teriparatide, and limiting weight bearing [4], while Bhattacharyya et al. reported successful treatment of incomplete PAFFs by only discontinuing BPs and limiting weight bearing [8]. Lee et al. reported preventive osteosynthesis surgery performed for a case in which femoral pain and fracture lines were revealed despite loading restriction and teriparatide administration for incomplete PAFFs [10]. Robinson et al. reported a comparative series of 10 cases of PAFFs and 12 cases of AFFs and found clinically significant differences in time to union, mortality, and complications. There was also a statistically significant difference in complications, i.e., in 12% of AFFs and 25% of PAFFs. Thus, Robinson et al. recommended prophylactic surgery for cases with clear fracture lines and/or cases with severe femoral pain [13]. However, the treatment may require a much more invasive procedure such as plate fixation or stem revision, since the key point in management of this fracture is recognition before a catastrophic displaced fracture occurs [3]. Strict conservative treatment such as non-weight-bearing exercises in hospitalization or prophylactic surgical treatment may be necessary because the conservative treatment was not effective in our cases.

The efficacy of teriparatide in conservative treatment for PAFFs has not been demonstrated, although teriparatide treatment was used in several case reports of PAFFs [3, 4, 10, 12]. Teriparatide is reported to significantly shorten postoperative recovery and reduce rates of delayed healing or nonunion in patients with AFFs, and AFFs and PAFFs are thought to have a similar pathogenic mechanism [21]. Furthermore, the efficacy of teriparatide for AFFs has been proposed in a systematic review [22].

Daily administration of teriparatide enhances bone healing more than weekly administration in complete AFF patients [23]. However, our patients were over 80 years old and their cognitive functions were declined; therefore, they could not self-administer teriparatide daily. Given the evidence that teriparatide may be useful, we chose to use teriparatide as part of postoperative treatment of PFFs in our two cases.

In the light of the limited amount of research on PAFFs, further studies are needed to determine if conservative or surgical treatment is best for incomplete PAFFs, the methods of appropriate conservative treatment, and the necessity of prophylactic surgical treatment to prevent complete PAFFs.

## Figures and Tables

**Table 1:** A review of the literature.

Author (s)/year	Number of cases	Age	Sex	Comorbidity	Use of BPs	Concomitant drug	Treatment	Teriparatide	Outcome
Sayed-Noor and Sjödén [2]/2009	1	78	F	None	+	None	Osteosynthesis	−	At 5 months under observation
Curtin and Fehring [3]/2011	3	Case 1: 52 Case 2: 85 Case 3: 79	All cases female	All cases had RA	All cases used	All cases used PSL	All cases conservative	Cases 2 and 3 used teriparatide	Cases 1 and 2, at 6 months under observation; case 3, 6 months taken for bone union
Cross et al. [4]/2012	1	81	F	Scoliosis	+		Conservative	+	At 6 months under observation
Schaeffer et al. [5]/2012	1	79	F	None	+		Stem revision	−	At 5 months under observation
Chen and Bhattacharyya [6]/2012	1	81	F	None	+		Osteosynthesis	−	At 10 weeks under observation
Reb et al. [7]/2013	1	74	F	None	+		Stem revision	−	At 6 months under observation
Bhattacharyya et al. [8]/2014	1	72	F	RA, Parkinson's disease	+	PSL	Conservative	−	At 3 months under observation
Niikura et al. [9]/2015	1	69	F	IP	+	PSL	Osteosynthesis	−	6 months taken for bone union
Lee et al. [10]/2015	3	Case 1: 43 Case 2: 74 Case 3: 86	All cases female	Case 1: SLE	All cases used	Case 1 used PSL	Case 1: osteosynthesis and needed reoperation; case 2: osteosynthesis; case 3: osteosynthesis	All cases used teriparatide	Case 1 11 months taken for bone union; case 2 at 7 months under observation; case 3 at 8 months under observation
Wakayama et al. [11]/2015	1	68	F	RA	+	PSL	Osteosynthesis	+	4 months taken for bone union
Woo et al. [12]/2016	1	82	F	None	+		Osteosynthesis	+	10 months taken for bone union
Robinson et al. [13]/2016	10	80	1 M; 9 F	None	All cases used		All operate	−	Average time to union was 8 months
Bottai et al. [14]/2017	1	77	F	Polymyalgia rheumatica	+	PSL	Osteosynthesis and needed reoperation	+	9 months taken for bone union

M: male; F: female; RA: rheumatoid arthritis; IP: interstitial pneumonia; SLE: systemic lupus erythematosus; BPs: bisphosphonates; PSL: prednisolone.
